# Integrated Multiomics Elucidates Molecular Mechanisms of Bisphenol A in Exacerbating Crohn’s Disease

**DOI:** 10.1155/mi/2903373

**Published:** 2026-02-12

**Authors:** Liangliang Dai, Chenjie Qiu

**Affiliations:** ^1^ Department of Urology, Wujin Hospital Affiliated With Jiangsu University, Changzhou, 213004, China, ujs.edu.cn; ^2^ Department of Urology, The Wujin Clinical College of Xuzhou Medical University, Changzhou, 213004, China, xzmc.edu.cn; ^3^ Department of General Surgery, Changzhou Hospital of Traditional Chinese Medicine, Changzhou, 213000, China, czzyy.com

**Keywords:** bisphenol a, Crohn’s disease, machine learning, molecular docking, network toxicology

## Abstract

**Background:**

Bisphenol A (BPA), a widespread environmental endocrine‐disrupting chemical, has been associated with the development and progression of Crohn’s disease (CD), yet its precise molecular mechanisms remain unclear. We aimed to systematically elucidate the potential molecular mechanisms by which BPA exacerbates CD and to identify key biomarkers and therapeutic targets.

**Methods:**

BPA‐related targets and CD transcriptomic datasets (GSE36807, GSE75214, and GSE95095) were retrieved from public databases. Overlapping genes were identified and subjected to functional enrichment and protein–protein interaction (PPI) network analysis. Multiple machine learning algorithms were employed to screen for core genes, and molecular docking was used to validate the binding affinity between BPA and core proteins. Immune infiltration analysis and regulatory network construction were performed to explore the roles of core genes in the immune microenvironment and post‐translational regulation.

**Results:**

A total of 65 overlapping genes between BPA and CD were identified, primarily enriched in pathways related to inflammation, apoptosis, and immune regulation. Machine learning screened five core genes (HGF, IL1R1, MMP1, MMP2, and NTRK2), which demonstrated strong diagnostic performance in independent datasets. Molecular docking revealed strong binding affinity between BPA and these proteins, with the lowest binding energy observed for MMP2 (−8.4 kcal/mol). Immune infiltration analysis indicated significant correlations between core genes and immune cell subsets such as Tr1, Th17, and Tfh cells. Regulatory network analysis identified key transcription factors (e.g., STAT3) and E3 ubiquitin ligases (e.g., SYVN1) involved in gene regulation.

**Conclusion:**

BPA may exacerbate CD progression by dysregulating core genes, such as HGF, IL1R1, MMP1, MMP2, and NTRK2, thereby disrupting inflammatory balance, extracellular matrix remodeling, and immune homeostasis.

**Summary**

• Identified 65 bisphenol A (BPA)–Crohn’s disease (CD) overlapping genes.
• Machine learning pinpointed five diagnostic core genes.
• Molecular docking revealed strong BPA‐core genes interaction.
• Immune infiltration highlighted immune–homeostasis disruption in CD.

## 1. Introduction

Bisphenol A (BPA), a synthetic compound extensively utilized in plastics and epoxy resins, is a pervasive environmental endocrine disruptor associated with a broad spectrum of health risks, including metabolic disorders, immune dysregulation, and inflammatory diseases [1–3]. BPA interferes with normal endocrine function by mimicking estrogen and disrupting its receptor‐binding processes [4]. Increasing evidence suggests that BPA is a significant risk factor for noncommunicable diseases, including inflammatory bowel disease (IBD) [5]. Notably, while BPA exposure has been implicated in exacerbating chronic inflammatory conditions, its precise molecular mechanisms in specific diseases, such as Crohn’s disease (CD), remain largely unexplored [6, 7]. CD is a relapsing‐remitting form of IBD driven by complex interactions among genetic susceptibility, immune dysregulation, and environmental factors [8–10]. Despite advancements in therapeutic strategies, the rising global incidence of CD underscores the urgent need to identify novel environmental risk factors and elucidate their pathophysiological mechanisms [11].

Epidemiological studies suggest that environmental influences contribute to CD risk through multiple pathways, including alterations in gut microbial composition, disruption of epithelial barrier integrity, immune modulation, and epigenetic modifications. For instance, the adoption of a Westernized diet—characterized by high intake of proteins, fats, and refined carbohydrates—correlates with increasing CD incidence [12]. Processed foods and emulsifiers may directly impair intestinal barrier function, while fiber deficiency reduces microbial diversity, promoting the expansion of pro‐inflammatory taxa [13, 14]. The hygiene hypothesis posits that reduced microbial exposure during early childhood—due to excessive hygiene, diminished contact with symbiotic microorganisms, and reduced parasite burden—may impair immune system development, predisposing individuals to aberrant immune responses against gut microbiota in adulthood [15–17]. Furthermore, early‐life exposure to broad‐spectrum antibiotics is strongly associated with microbiota dysbiosis, potentially disrupting immune tolerance and predisposing individuals to CD [18, 19]. Environmental toxicants, such as airborne particulate matter (e.g., PM2.5) and microplastics, may also contribute to disease pathogenesis through oxidative stress and immune activation pathways [20, 21]. Among environmental risk factors, tobacco smoking remains the most well‐established contributor to CD progression, exacerbating inflammation by altering intestinal blood flow, mucus production, and immune cell activity [22, 23]. Recent studies indicate that BPA significantly influences systemic inflammatory responses in CD patients, particularly those with compromised intestinal barrier function and dysregulated bacterial secretion products in the bloodstream [24]. However, systematic investigations into how BPA interacts with CD‐associated molecular networks remain limited. The advent of multiomics databases, machine learning, and computational biology offers unprecedented opportunities to dissect these interactions at a systems level.

In this study, we aimed to elucidate the molecular interplay between BPA exposure and CD pathogenesis by integrating bioinformatics, machine learning, and structural biology approaches. We hypothesized that BPA perturbs key signaling pathways and immune responses in CD through interactions with hub genes, thereby increasing disease susceptibility. By systematically identifying overlapping targets, validating core regulatory elements, and mapping their functional networks, this work seeks to bridge the existing knowledge gap between environmental toxicology and CD etiology, ultimately identifying potential biomarkers and therapeutic targets to mitigate BPA‐associated disease risks.

## 2. Materials and Methods

### 2.1. Data Collection and Target Identification

We retrieved the 2D and 3D chemical structures of BPA, along with its SMILES notation, from the PubChem database (https://pubchem.ncbi.nlm.nih.gov/) to facilitate accurate identification of potential targets [25]. BPA‐related targets were then obtained from three databases: ChEMBL (https://www.ebi.ac.uk/chembl/), STITCH (http://stitch.embl.de/), and SwissTargetPrediction (http://swisstargetprediction.ch/) [26–28]. After removing duplicates, a final list of BPA targets was compiled. For CD‐related data, transcriptomic datasets (GSE36807, GSE75214, and GSE95095) were downloaded from the GEO database (https://www.ncbi.nlm.nih.gov/geo/). All datasets (GSE36807, GSE75214, and GSE95095) were subjected to log_2_ transformation and normalization using the normalizeBetweenArrays function in the limma package to ensure within‐dataset comparability. When integrating these datasets, batch effects were corrected using the ComBat function from the sva package [29]. The effectiveness of correction was assessed via principal component analysis (PCA) and boxplot visualization. Differential expression analysis was conducted using the “limma” package, applying thresholds of |log_2_ fold change| >1 and an adjusted *p*‐value <0.05 [30]. Additionally, CD‐associated targets were extracted from GeneCards (https://www.genecards.org/, score ≥ 20), OMIM (https://www.omim.org/), and TTD databases (https://db.idrblab.net/ttd/) [31–33]. Merging differentially expressed genes (DEGs) with database‐derived targets yielded a comprehensive set of CD‐associated genes. Overlapping BPA and CD targets were then identified using a Venn diagram.

### 2.2. Functional Enrichment and Network Analysis

Gene Ontology (GO) and Kyoto Encyclopedia of Genes and Genomes (KEGG) enrichment analyses were performed using the “clusterProfiler” package in R, applying significance thresholds of *p*  < 0.05 and false discovery rate (FDR) < 0.05. Gene co‐expression networks for BPA‐induced CD targets were generated using GeneMANIA (https://genemania.org/) [34]. Protein–protein interaction (PPI) networks were constructed via STRING (https://cn.string-db.org/) and analyzed in Cytoscape [35]. Hub genes were identified using the CytoHubba plugin, employing betweenness, closeness, and degree algorithms, with only the top 20 ranked genes across all three methods retained.

### 2.3. Machine Learning–Based Screening

A total of 27 candidate genes were evaluated using a machine learning workflow. The training set, comprising integrated datasets (GSE36807, GSE75214, and GSE95095), and the external validation sets (GSE102133 and GSE179285) were preprocessed using quantile normalization. Ten machine learning algorithms—Lasso, SVM, RF, glmBoost, Stepglm, Ridge, Enet, GBM, LDA, XGBoost, and NaiveBayes—were applied across 113 machine learning models [36]. Model performance was assessed using area under the curve (AUC), accuracy, precision, recall, F1‐score, and false positive rate (FPR). Core genes were identified based on feature importance scores from the optimal model.

### 2.4. Molecular Docking

The 3D structures of HGF, IL1R1, MMP1, MMP2, and NTRK2 were retrieved from the Protein Data Bank database (https://www.rcsb.org/) [37], while the structure of BPA (CID: 6623) was obtained from PubChem. CB‐Dock2 (https://cadd.labshare.cn/cb-dock2/index.php), a blind docking tool for computer‐aided drug discovery, predicts binding sites and affinities by analyzing the structural compatibility between protein receptors and small‐molecule ligands [38].

### 2.5. Immune Infiltration Analysis

The ImmuCellAI platform (http://bioinfo.life.hust.edu.cn/ImmuCellAI/) was used to quantify 24 immune cell types in the training dataset [39]. Differences between the CD and control groups were analyzed using the Wilcoxon test. Spearman correlation analysis was performed to assess the relationship between core gene expression and immune cell infiltration levels.

### 2.6. Regulatory Network Construction

A multilayered regulatory network encompassing PPIs, transcription factors, miRNAs, drugs, and chemicals was constructed using the GenDoma platform (https://ai.citexs.com/homePath). The UbiBrowser database (http://ubibrowser.bio-it.cn/ubibrowser/) was utilized to analyze interactions between known and predicted human ubiquitin ligases (E3) and their substrates. Key genes were further examined to identify essential E3 ubiquitin ligases that may be affected using UbiBrowser [40].

## 3. Results

### 3.1. Identification and Integration of BPA‐ and CD‐Associated Targets

Initially, we retrieved 647, 10, and 39 BPA‐associated targets from the ChEMBL, STITCH, and SwissTargetPrediction databases, respectively. After removing duplicates, we identified 690 unique BPA targets (Figure 1A). To identify CD‐related targets, we utilized transcriptomic datasets (GSE36807, GSE75214, and GSE95095) from the GEO database. Advanced data normalization techniques and batch effect correction were applied to integrate these datasets into a unified training set. The effectiveness of batch correction was assessed using PCA and box plots, which demonstrated a significant reduction in batch effects (Figure 1B,C). Differential expression analysis of the training set identified 1356 DEGs, including 674 upregulated and 682 downregulated genes in CD (Figure 1D). A heatmap was generated to visualize the top 50 upregulated and downregulated genes with the most significant fold changes (Figure 1E). Next, we retrieved 618, 140, and 34 CD‐related targets from the GeneCards, OMIM, and TTD databases, respectively. After merging these targets with the DEGs and eliminating duplicates, we obtained a total of 1954 CD‐related genes (Figure 1F). Finally, a Venn diagram analysis identified 65 CD targets associated with BPA, highlighting their potential role in the molecular mechanisms through which BPA may contribute to CD risk (Figure 1G).

Figure 1Integrated analysis of multi‐database target prediction. (A) Venn diagram illustrating the target genes of BPA among the ChEMBL, STITCH, and SwissTargetPrediction databases. (B) PCA plots comparing data before and after batch correction for three GEO datasets. (C) Boxplots comparing data before and after batch correction for three GEO datasets. (D) Volcano plot of DEGs between control and treat groups. (E) Heatmap of top 50 upregulated and downregulated genes in the treat group. (F) Venn diagram analyzing target overlaps among GeneCards, OMIM, TTD, and DEGs. (G) Venn diagram showing the overlap genes between BPA and CD targets.

**Figure:**
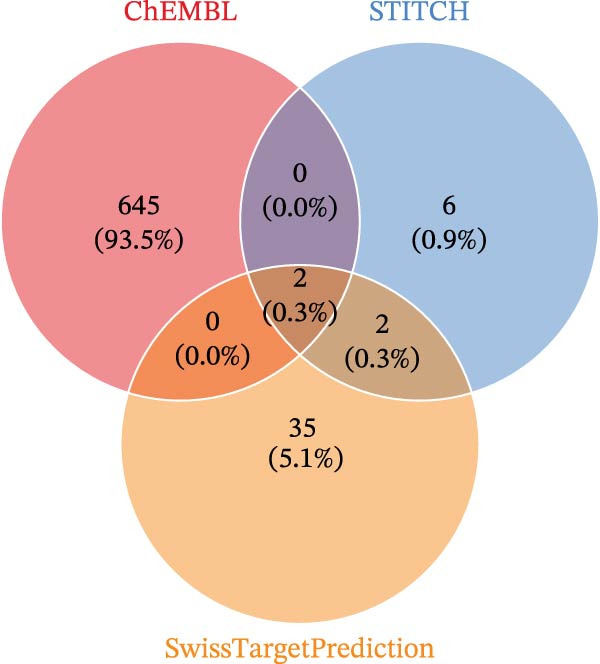
(A)

**Figure:**
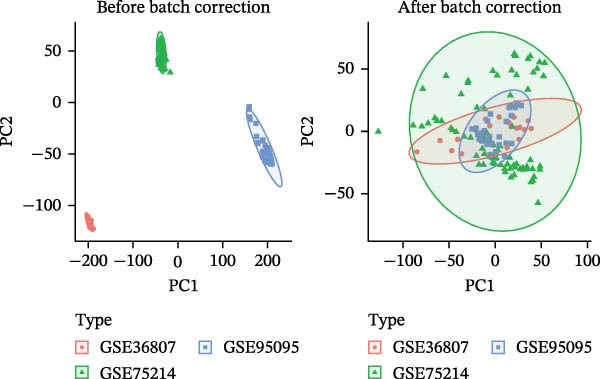
(B)

**Figure:**
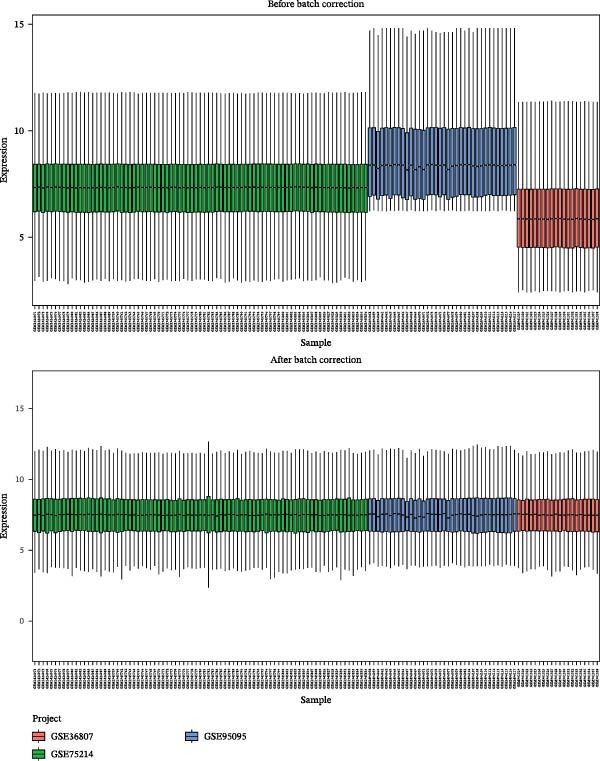
(C)

**Figure:**
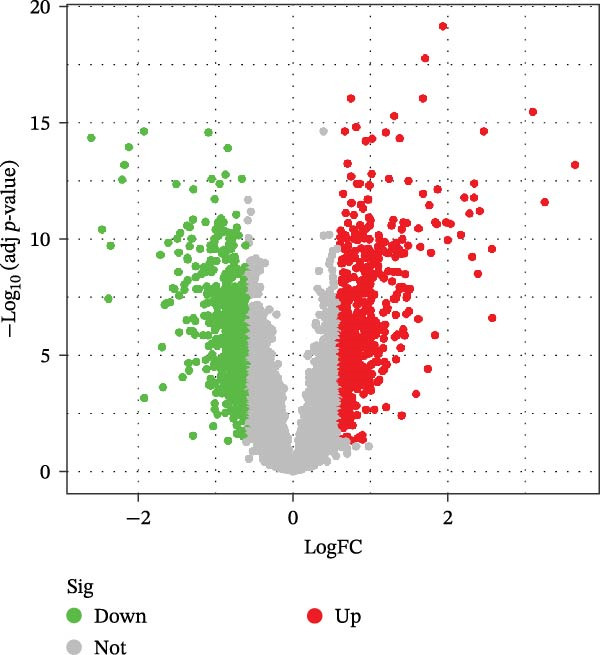
(D)

**Figure:**
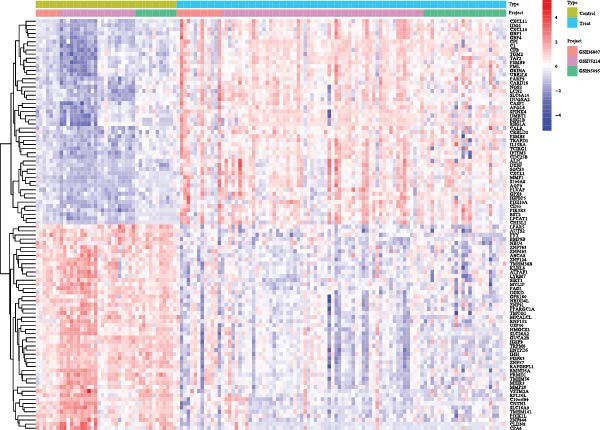
(E)

**Figure:**
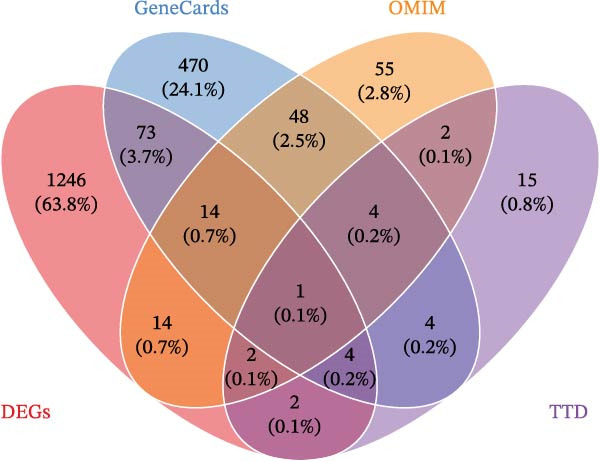
(F)

**Figure:**
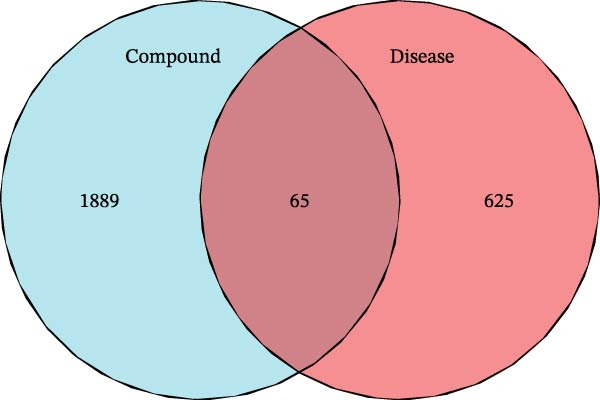
(G)

### 3.2. Functional Enrichment and Network Analysis of BPA–CD Target Genes

We then conducted GO and KEGG enrichment analyses on the 65 potential targets identified earlier. GO analysis revealed that these genes are primarily involved in biological processes (BPs) such as apoptosis, signal transduction regulation, inflammation, stress response, and MAPK cascade activation. In terms of cellular components (CCs), these genes are associated with cell membrane structures and organelle compartments. Regarding molecular functions (MFs), they are linked to receptor and binding activities, phosphodiesterase enzymatic activity, as well as metallopeptidase and metalloendopeptidase functions (Figure 2A). KEGG pathway analysis indicated that these genes are enriched in key signaling pathways, including apoptosis, focal adhesion, VEGF, HIF‐1, TNF, cAMP, cGMP‐PKG, Ras, calcium signaling, PI3K‐Akt, and MAPK pathways. Additionally, they are implicated in various metabolic processes, such as sphingosine metabolism, purine metabolism, glycosaminoglycan degradation, and nitrogen metabolism (Figure 2B). Next, we constructed gene co‐expression and PPI networks using the GeneMANIA and STRING databases, respectively. The gene co‐expression network highlighted functional associations with phosphodiesterase activity, metallopeptidase activity, and ligand‐activated transcription factors (Figure 2C). The PPI network, consisting of 65 nodes and 236 edges, provided insights into the interactions among these genes (Figure 2D). To prioritize key genes, we applied the CytoHubba algorithm, ranking the top 20 genes based on betweenness, closeness, and degree centrality measures (Figure 2E–G). Among these, 13 genes were consistently identified across all three ranking methods. Ultimately, a final set of 27 key genes was selected for further analysis (Figure 2H).

Figure 2Functional enrichment and network analysis of 65 common DEGs for BPA and CD. (A) Bar chart illustrating GO enrichment results. Blue, red, and green bars represent BP, CC, and MF, respectively. (B) A bubble plot of KEGG pathway enrichment. (C) Interaction networks for 65 DEGs using GeneMANIA database. (D) PPI network for proteins encoded by the above DEGs according to STRING database. Subnetworks centered on key genes calculated by three algorithms like betweenness (E), closeness (F), and degree (G). (H) Venn diagram analyzing overlaps of topological metrics (betweenness, closeness, and degree).

**Figure:**
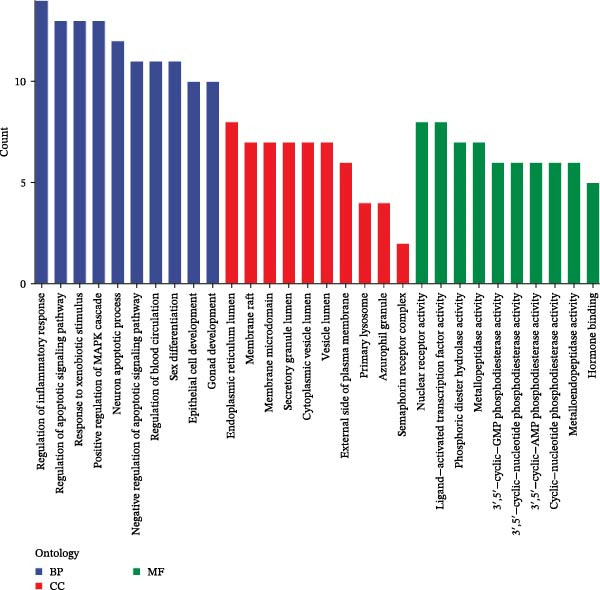
(A)

**Figure:**
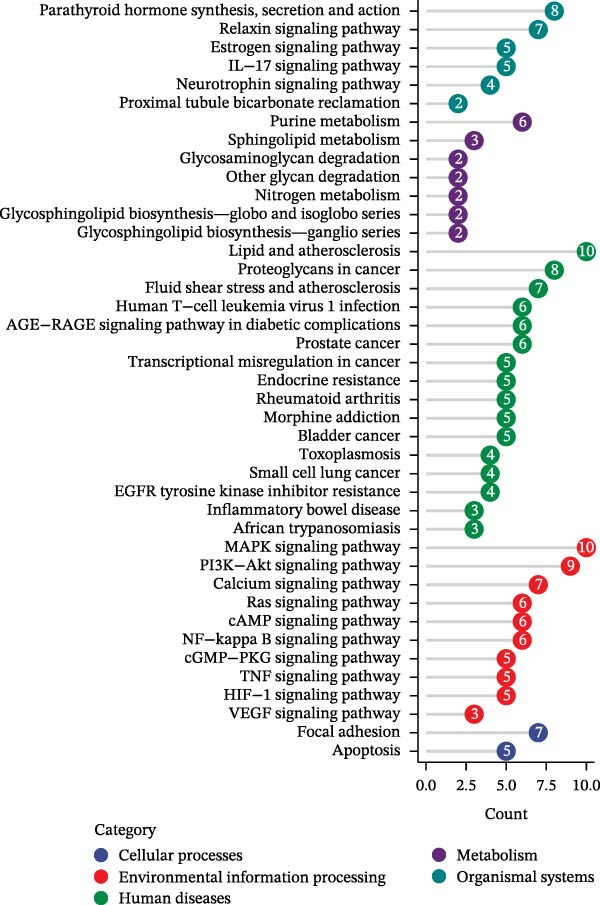
(B)

**Figure:**
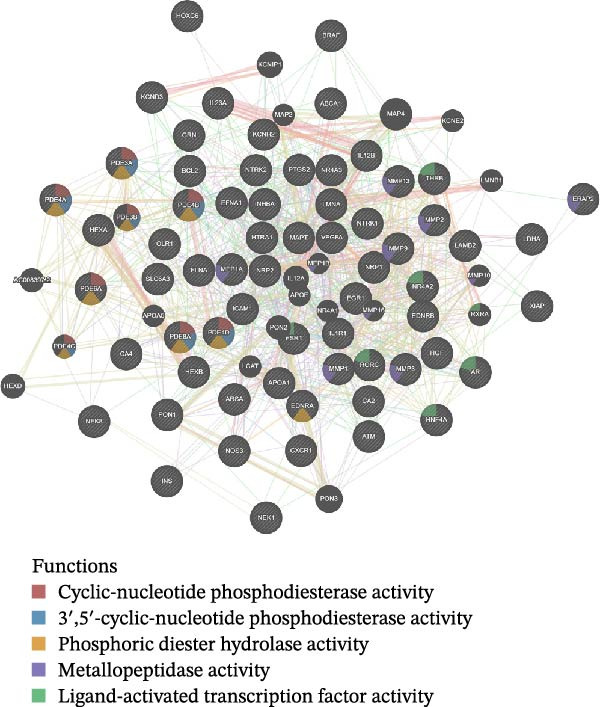
(C)

**Figure:**
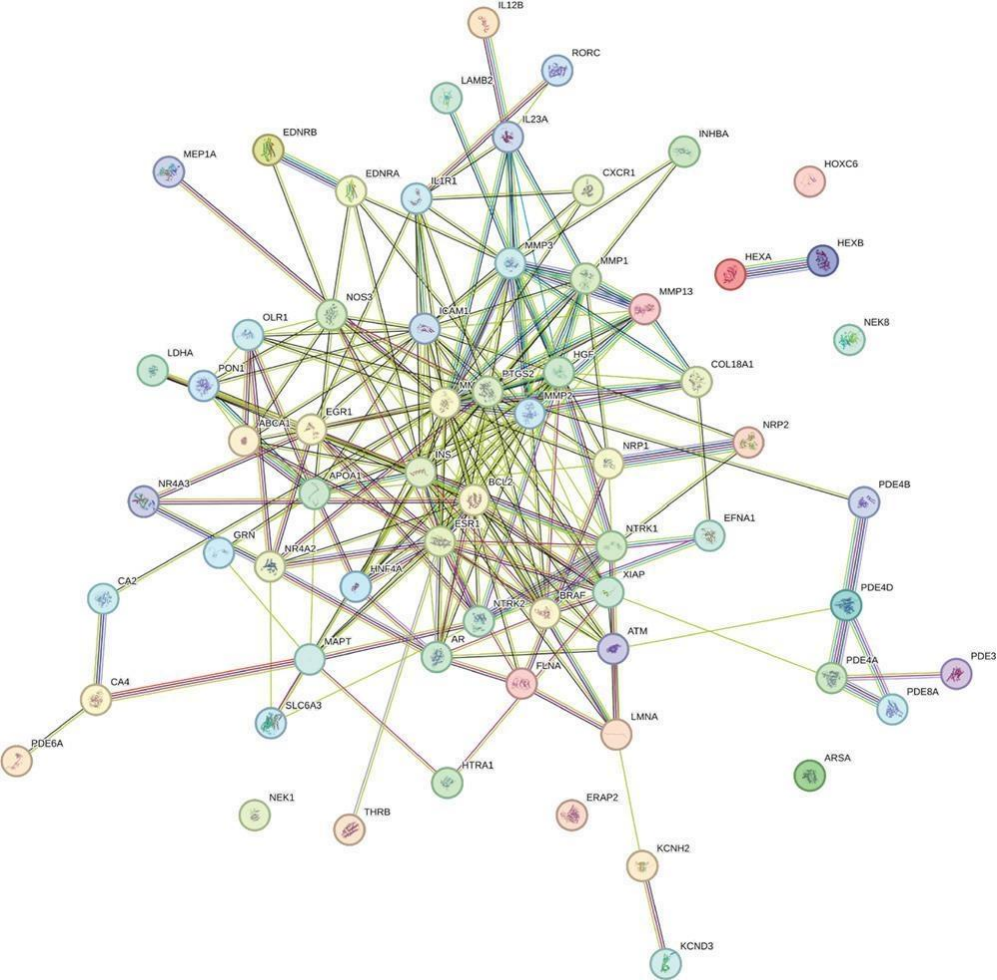
(D)

**Figure:**
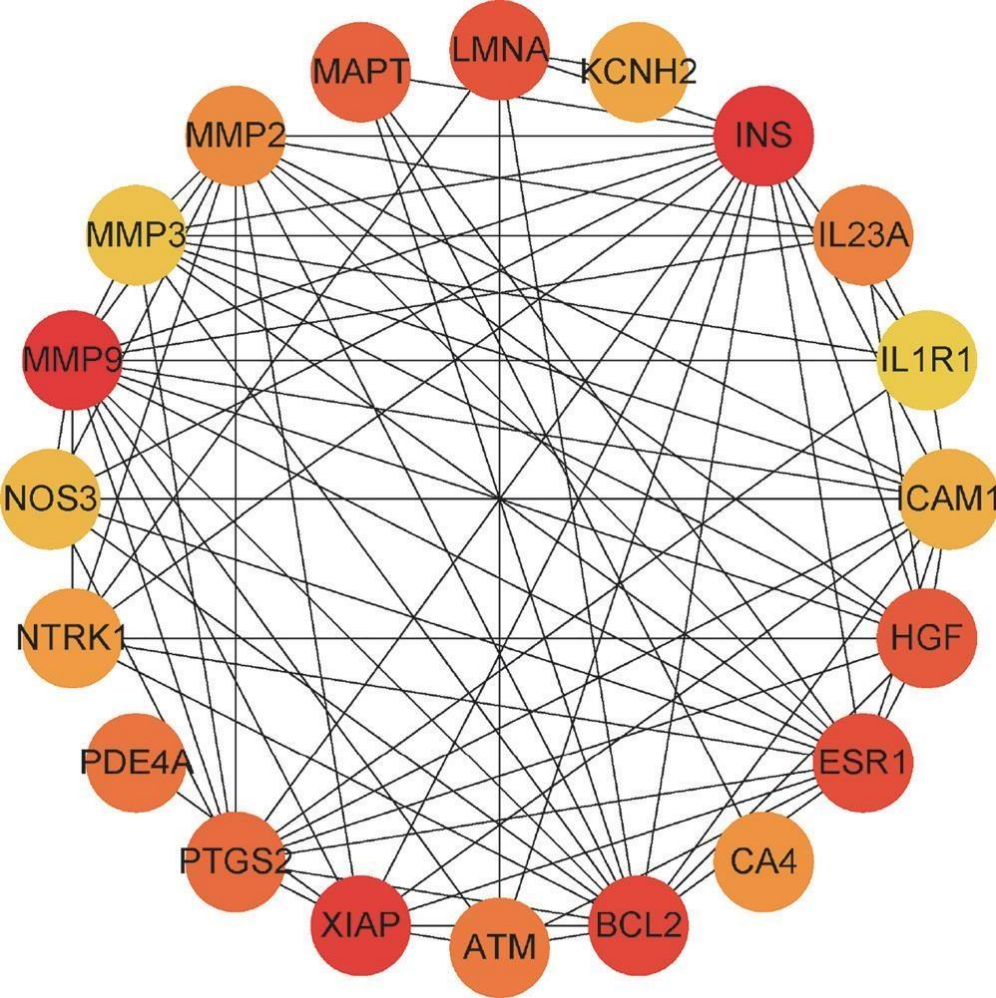
(E)

**Figure:**
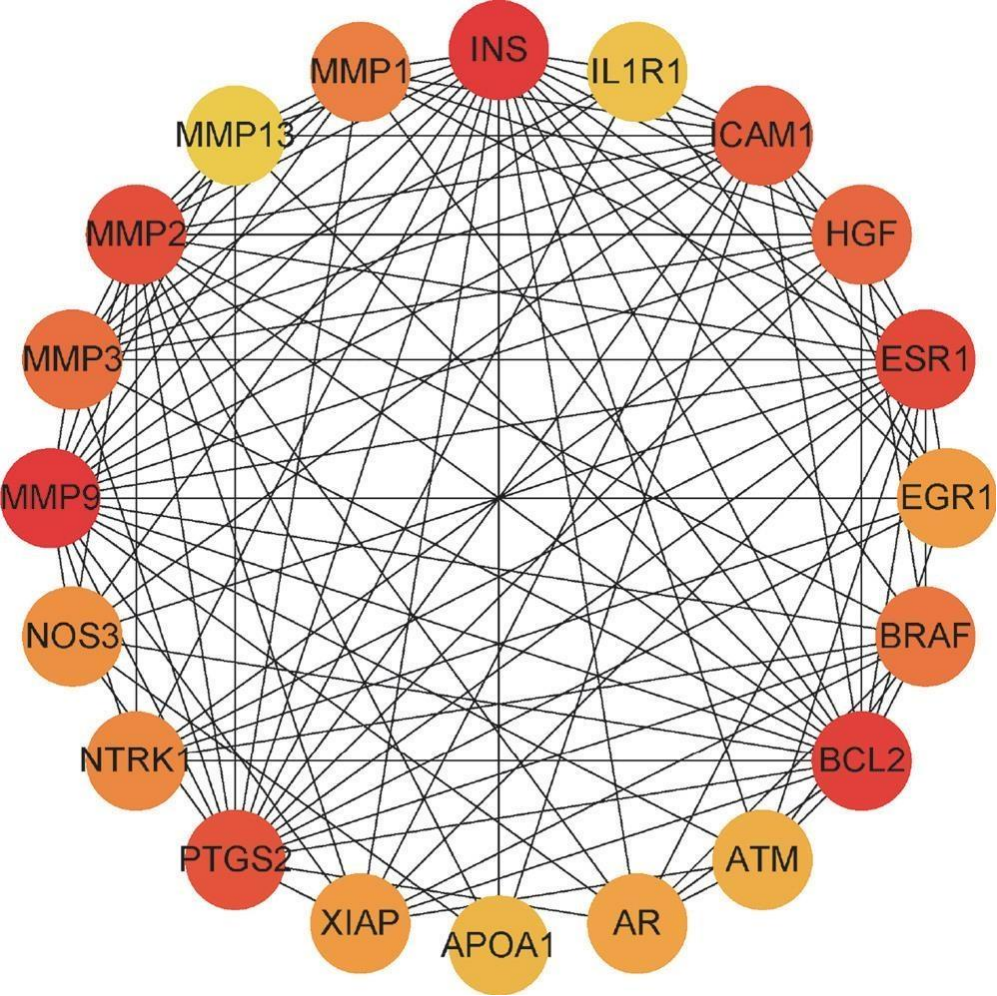
(F)

**Figure:**
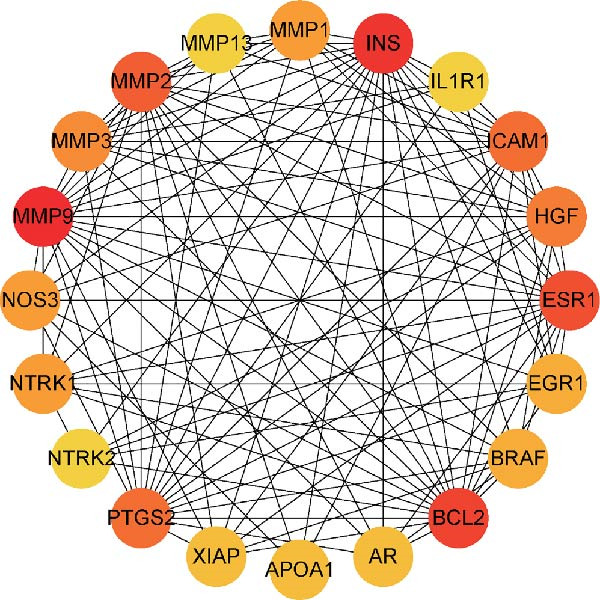
(G)

**Figure:**
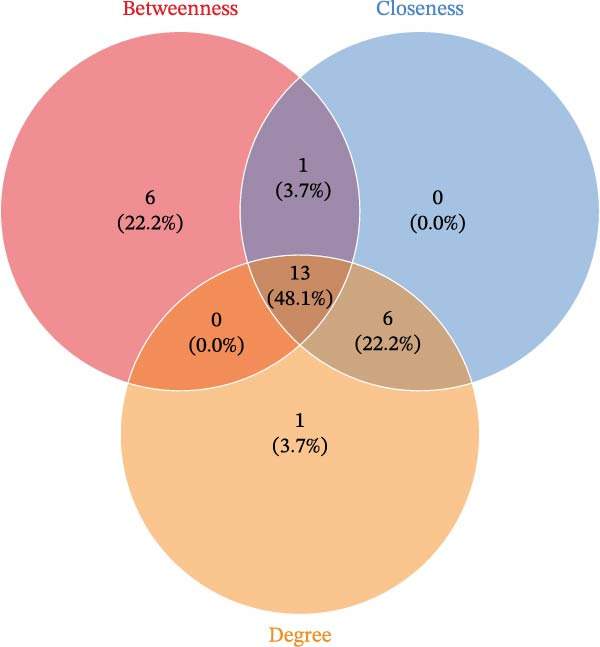
(H)

### 3.3. Machine Learning–Based Identification and Validation of Core Genes

To identify core genes associated with CD, we screened and validated 27 candidate genes using an integrated machine learning workflow across both training and external validation datasets. The glmBoost + GBM model demonstrated the highest predictive performance, achieving an average AUC of 0.974 across all datasets (Figure 3A). Specifically, the model attained AUC values of 0.999 in the training set, 0.951 in GSE102133, and 0.972 in GSE179285 (Figure 3B). Confusion matrix analysis confirmed a strong concordance between predicted and actual classifications for both control and disease groups (Figure 3C). In the training set, the model achieved an accuracy of 0.9781, precision of 0.9756, recall of 0.9524, F1‐score of 0.9639, and FPR of 0.0105 (Figure 3D). Although recall was slightly lower in the GSE102133 validation set, the model demonstrated robust performance in GSE179285, with accuracy, precision, recall, and F1‐score all exceeding 0.85 (Figure 3D). Using this model, we identified 10 core genes, revealing significant correlations among them, particularly a strong positive correlation between IL1R1 and MMP2 (Figure 3E). We then compared the expression levels and diagnostic potential of these core genes across the training set, GSE102133, and GSE179285. MMP1, HGF, IL1R1, and MMP2 were significantly upregulated in CD, while NTRK2 was downregulated (Figure 3F–H). Diagnostic performance analysis showed that the AUC values of these core genes exceeded 0.75 in both the training set and GSE102133 (Figure 3I, J), and surpassed 0.8 in GSE179285 (Figure 3K).

Figure 3Comprehensive analysis of model performance and gene expression patterns. (A) Summary of test efficacy for diverse machine learning models. (B) ROC curves for glmBoost + GBM model in the training set, GSE102133, and GSE179285. (C) Confusion matrix for the training set, GSE102133, and GSE179285. (D) Performance metrics, like accuracy, precision, recall, and F1 score, are compared across the training set and validation datasets (GSE102133 and GSE179285). (E) Visualizes pairwise correlations between 10 model genes. Boxplots comparing expression levels of 10 model genes between control and treat groups in the training set (F), GSE102133 (G), and GSE179285 (H). Multigroup ROC curves for 10 model genes in the training set (I), GSE102133 (J), and GSE179285 (K).  ^∗^ *p* < 0.05;  ^∗∗^ *p* < 0.01;  ^∗∗∗^ *p* < 0.001.

**Figure:**
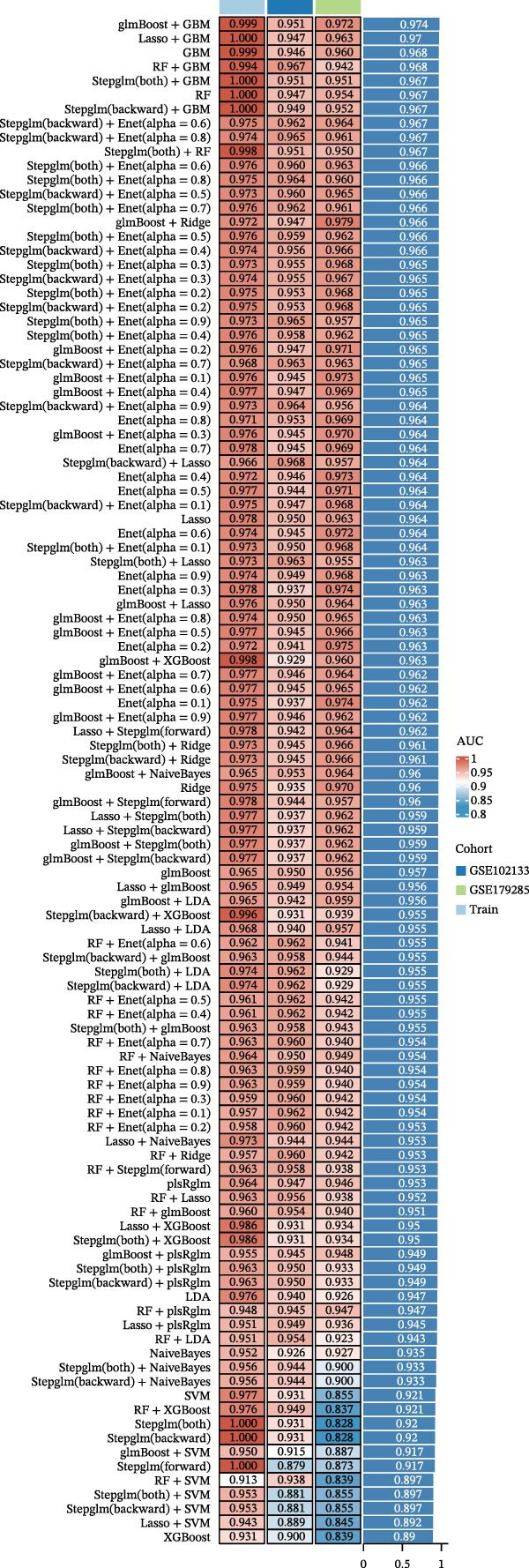
(A)

**Figure:**
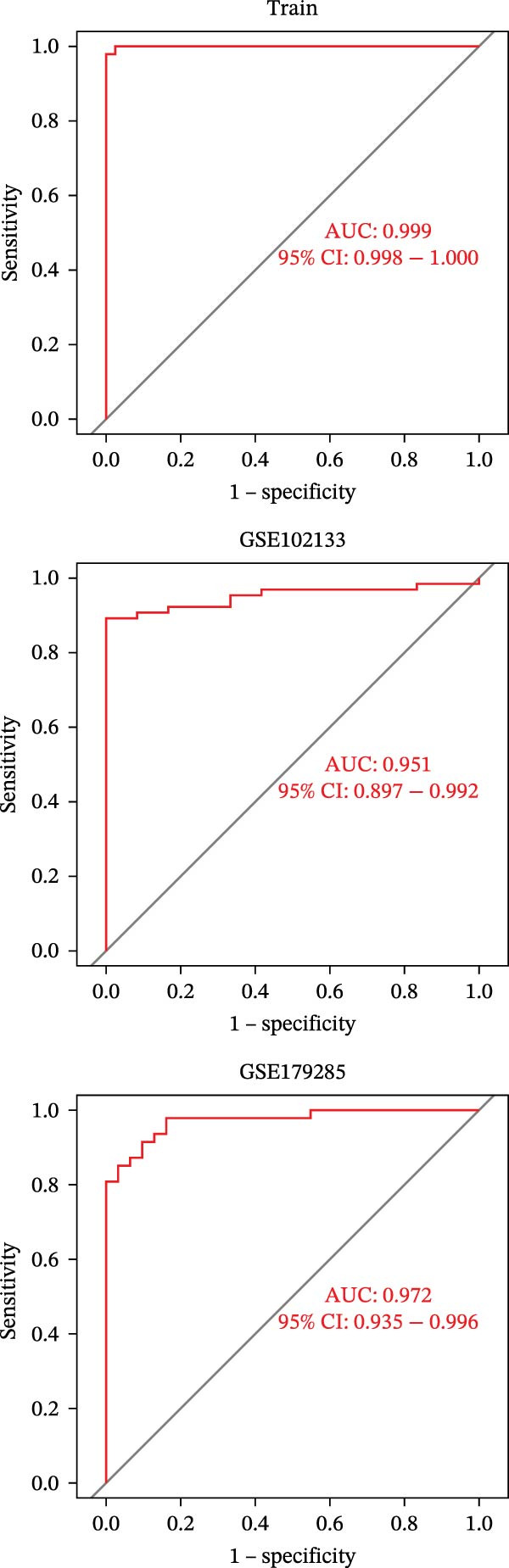
(B)

**Figure:**
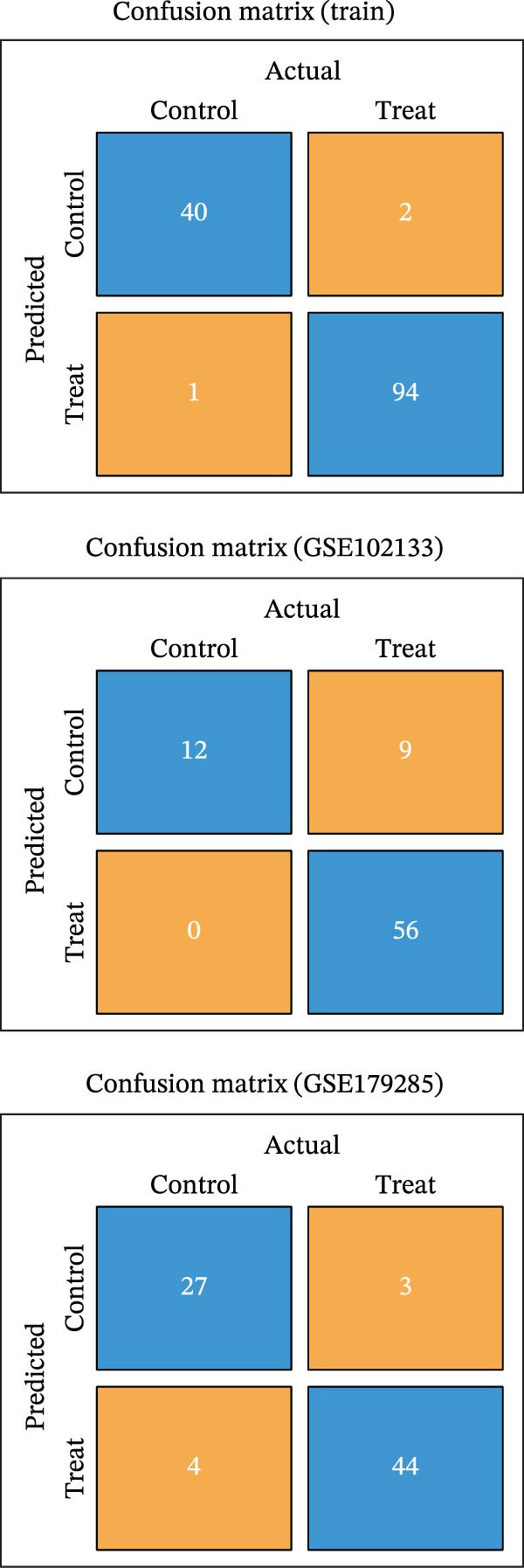
(C)

**Figure:**
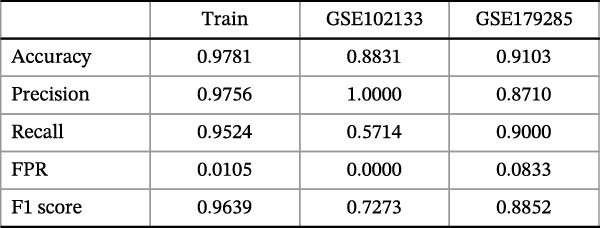
(D)

**Figure:**
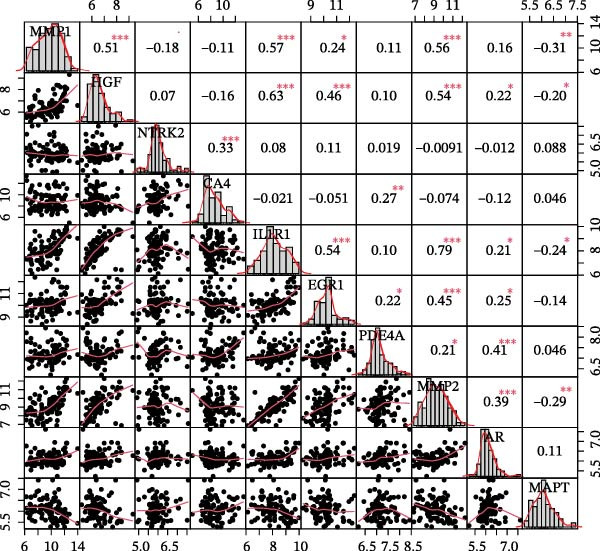
(E)

**Figure:**
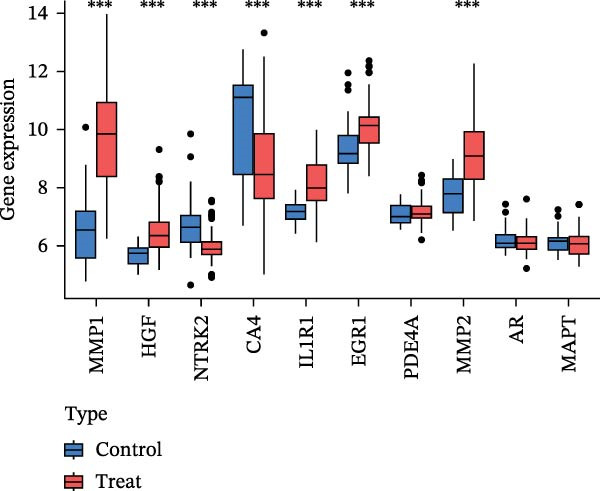
(F)

**Figure:**
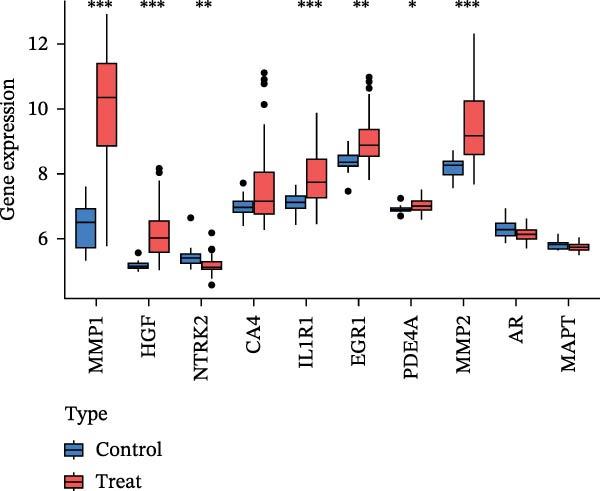
(G)

**Figure:**
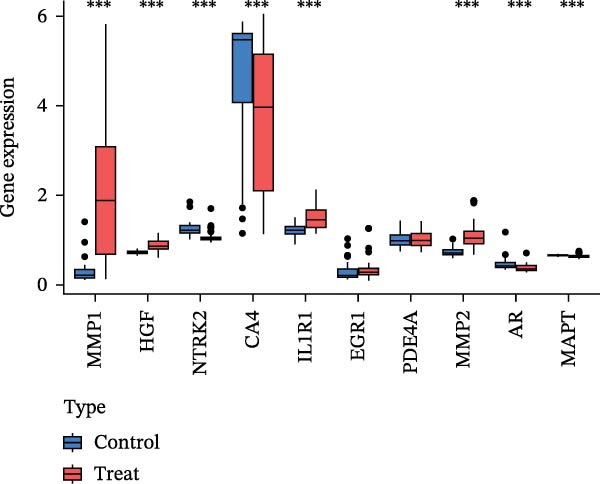
(H)

**Figure:**
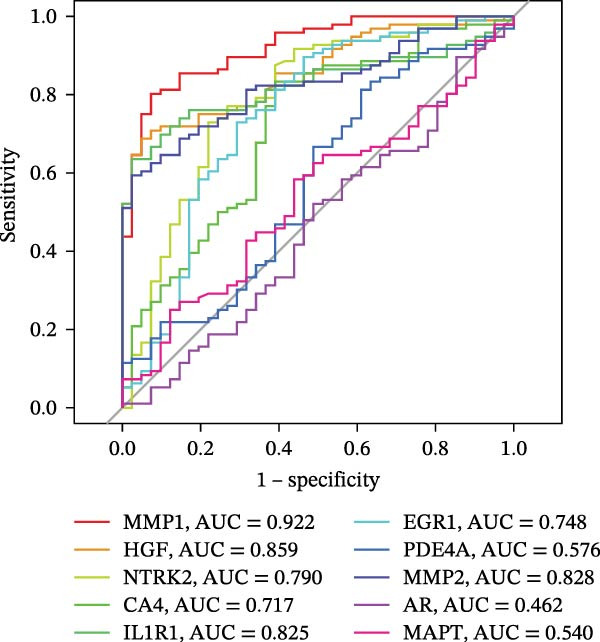
(I)

**Figure:**
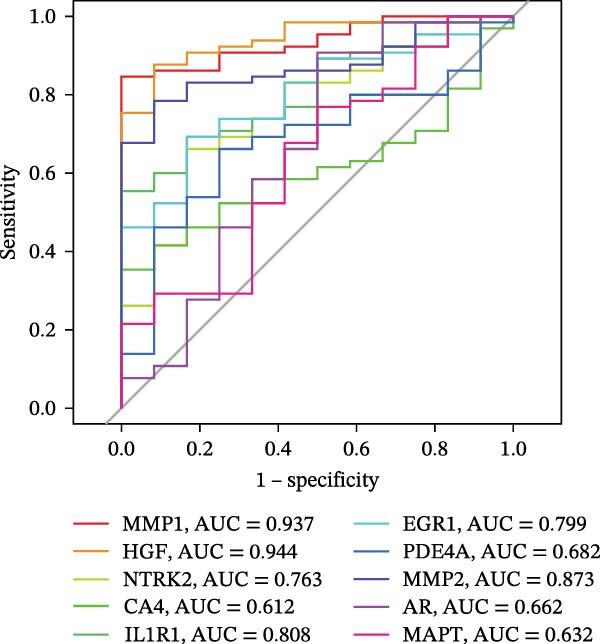
(J)

**Figure:**
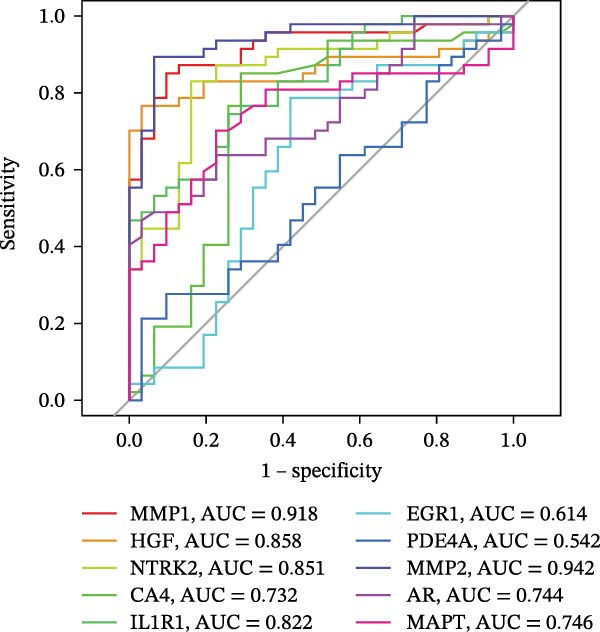
(K)

### 3.4. Molecular Docking Analysis of Core Genes

Through screening of training and validation datasets, we identified HGF, IL1R1, MMP1, MMP2, and NTRK2 as key genes mediating the increased risk of CD induced by BPA exposure. Molecular docking analysis revealed the binding affinities (Vina scores) between BPA and these genes as follows: HGF (−6.0 kcal/mol), IL1R1 (−7.8 kcal/mol), MMP1 (−7.3 kcal/mol), MMP2 (−8.4 kcal/mol), and NTRK2 (−7.2 kcal/mol). Structural visualization was further performed using the CB‐DOCK2 database (Figure 4A–E). Based on the criterion of strong binding affinity (Vina score ≤ −7.0 kcal/mol), IL1R1, MMP1, MMP2, and NTRK2 exhibited robust interactions with BPA. Notably, MMP2 showed the strongest binding affinity, suggesting its potential role as a critical mediator in BPA‐induced CD pathogenesis.

Figure 4Molecular docking visualization and analysis of compound‐protein interactions. The five core proteins are HGF (A), IL1R1 (B), MMP1 (C), MMP2 (D), and NTRK2 (E).

**Figure:**
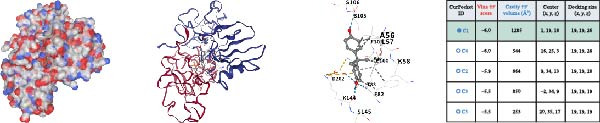
(A)

**Figure:**
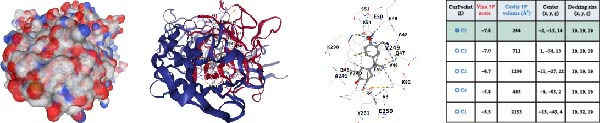
(B)

**Figure:**
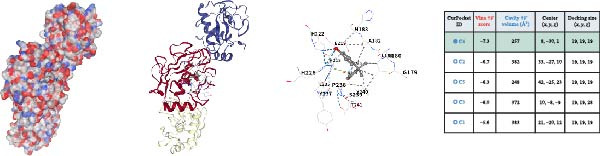
(C)

**Figure:**
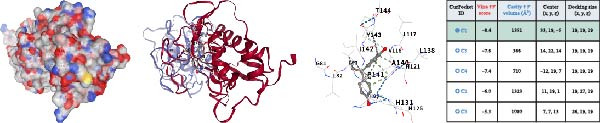
(D)

**Figure:**
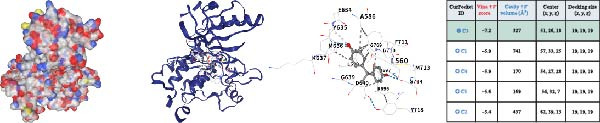
(E)

### 3.5. Immune Infiltration Analysis and Correlation With Core Genes

Using the ImmuCellAI platform, we performed immune infiltration analysis on the training dataset to evaluate the distribution of 24 immune cell types (Figure 5A). Comparisons between the control and treatment groups revealed significant differences in 14 immune cell subsets. Specifically, NK cells, CD4 T cells, CD4 naïve T cells, Tr1, nTreg, iTreg, Th17, Tfh, and exhausted T cells showed higher infiltration in the treatment group, while B cells, CD8 T cells, CD8 naïve T cells, cytotoxic T cells, and effector memory T cells were more abundant in the control group (Figure 5B). Correlation analysis between the five core genes and immune cell populations revealed distinct association patterns. HGF, IL1R1, MMP1, and MMP2 were positively correlated with Tr1, Th17, Tfh, nTreg, CD4 T cells, CD4 naïve T cells, and macrophages, while showing negative correlations with monocytes, CD8 T cells, CD8 naïve T cells, gamma delta T cells, cytotoxic T cells, and effector memory T cells (Figure 5C–F). In contrast, NTRK2 exhibited positive associations with central memory T cells, Th1 cells, and CD4 naïve T cells, but negative correlations with monocytes and NK cells (Figure 5G).

Figure 5Immune infiltration analysis for CD and the relationship between core genes and immune cells. (A) Heatmaps display the immune microenvironment of 24 immune cells for control and treat samples. (B) Boxplots compare the infiltration proportions of immune cells between the control and treat groups. The relationship between 24 types of immune cells and HGF (C), IL1R1 (D), MMP1 (E), MMP2 (F), and NTRK2 (G).

**Figure:**
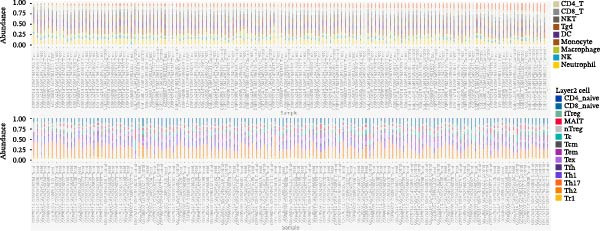
(A)

**Figure:**
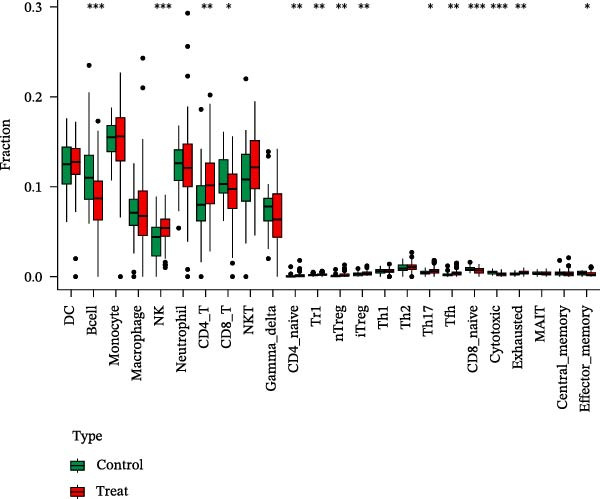
(B)

**Figure:**
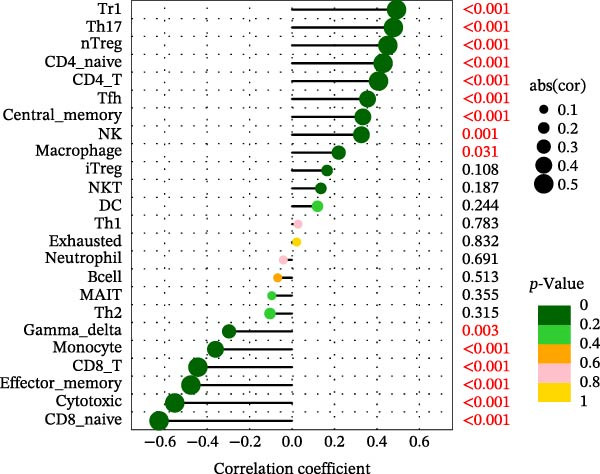
(C)

**Figure:**
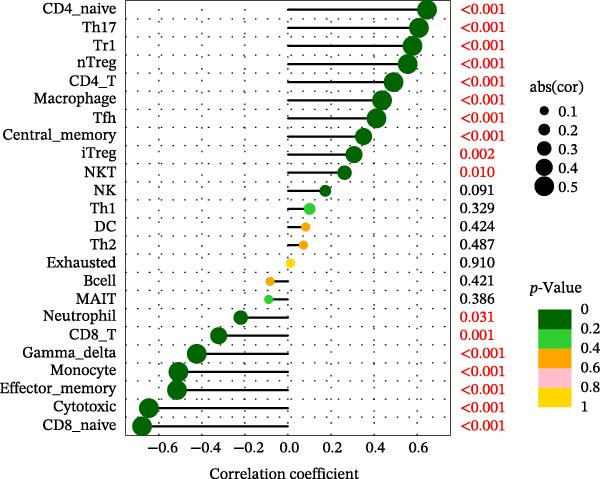
(D)

**Figure:**
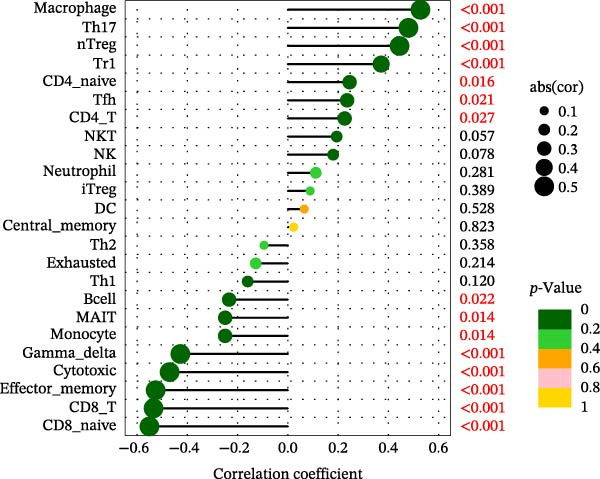
(E)

**Figure:**
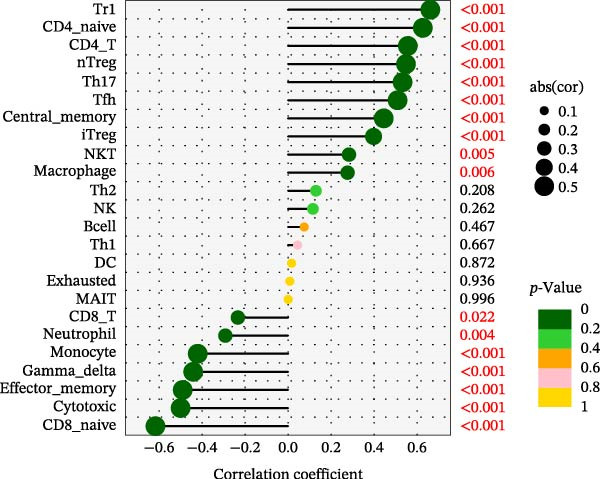
(F)

**Figure:**
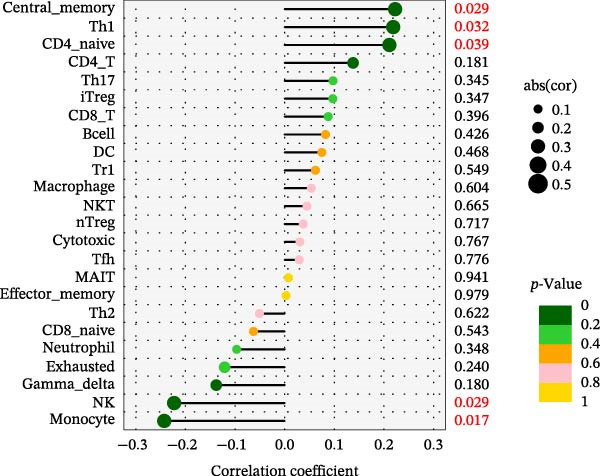
(G)

### 3.6. Comprehensive Regulatory Network of Core Genes

To further investigate the regulatory mechanisms underlying the five core genes, we constructed a comprehensive regulatory network for HGF, IL1R1, MMP1, MMP2, and NTRK2. This network integrated PPI, transcription factor–target gene relationships, miRNA–target gene associations, drug–target interactions, and chemical–gene interactions (Figure 6A). Network analysis revealed STAT3, COL3A1, and CCL2 as key interacting proteins for HGF, IL1R1, MMP1, and MMP2, while MAZ was identified as a critical transcription factor. Additionally, several key compounds interacting with these genes were highlighted, including progesterone, cisplatin, estradiol, doxorubicin, smoke‐related agents, quercetin, hydrogen peroxide, dexamethasone, particulate matter, and benzo(a)pyrene (Figure 6B). A systematic analysis of E3 ubiquitin ligases associated with the core genes identified the top 20 most strongly linked ligases (Figure 6C). Among these, SYVN1, NEDD4, STUB1, CBLC, and SMURF1 emerged as prominent E3 ubiquitin ligases interacting with the core genes, suggesting their potential role in post‐translational regulation.

Figure 6Molecular interaction network analysis for five core genes. (A) Networks, including PPI, transcription factors, miRNAs, drugs, and chemicals for five core genes using the GenDoma platform. (B) Common network node of five core genes. (C) Networks between E3 ubiquitin ligases and five core genes using UbiBrowser database.

**Figure:**
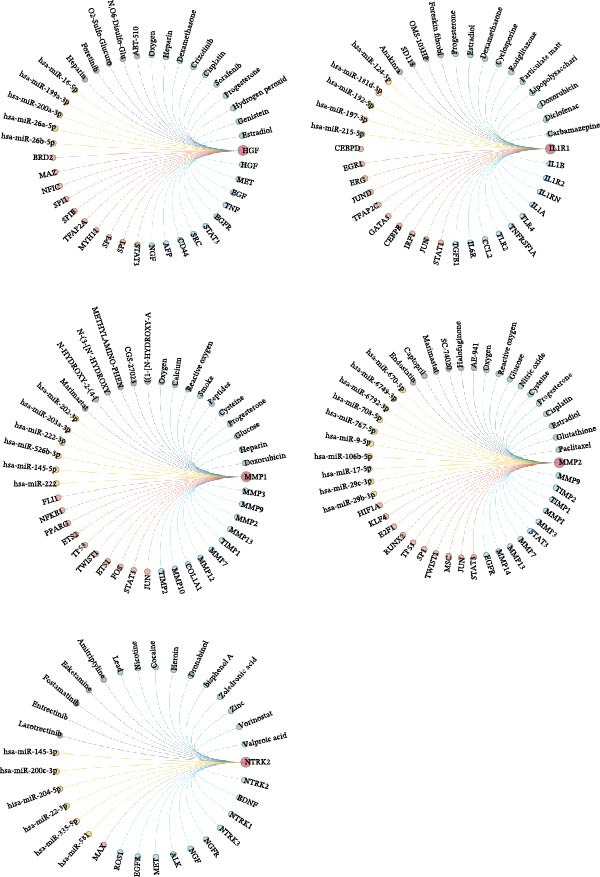
(A)

**Figure:**
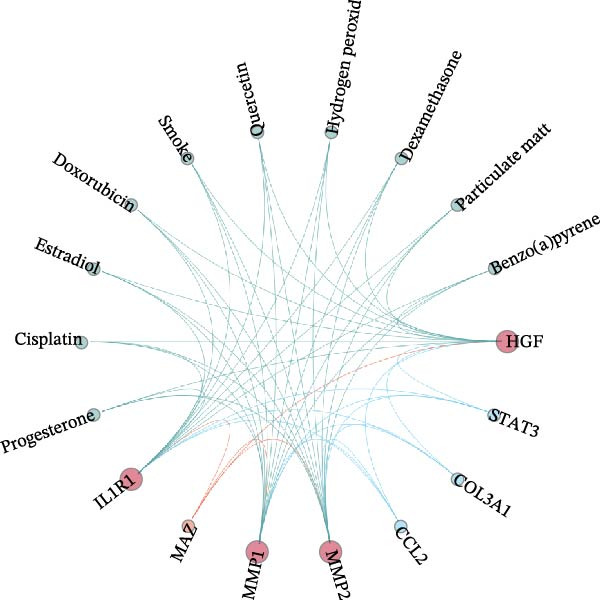
(B)

**Figure:**
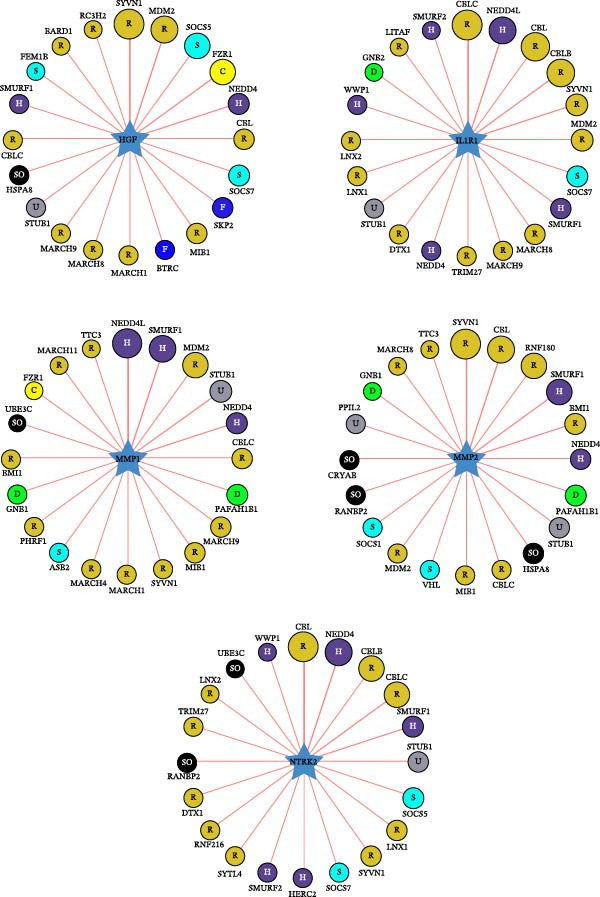
(C)

## 4. Discussion

Previous studies have demonstrated that BPA exposure exacerbates gastrointestinal chronic inflammation, a hallmark of IBD, by altering gut microbiota composition, increasing intestinal permeability, and promoting immune cell activation [41]. Using integrated approaches, such as network toxicology, PPI analysis, and molecular docking, Hong et al. [2] identified that BPA accelerates IBD progression by upregulating CCND1 expression. Additionally, BPA and its substitutes induce dysregulation of glucose and fatty acid metabolism in ulcerative colitis (UC), with their synergistic effects on intestinal metabolic pathways promoting UC progression [42]. However, research on the association between BPA and CD remains limited. An observational study found significantly elevated serum BPA levels in patients with active CD compared to those in remission, and in colonic CD compared to ileal CD [24]. BPA exposure compromises intestinal barrier integrity, facilitating bacterial and endotoxin translocation into systemic circulation, which subsequently triggers widespread immune responses. Moreover, BPA perturbs gut microbiota composition, suppressing the production of immunomodulatory metabolites (e.g., butyrate and tryptophan) and disrupting intestinal immune homeostasis [43, 44]. BPA also promotes aberrant secretion of pro‐inflammatory cytokines, such as IL‐23 and IL‐17, exacerbating chronic inflammation through hyperactivation of Th17 cells [45, 46]. Building on these findings, this study focuses on CD, utilizing multiomics strategies that integrate compound‐disease databases, cross‐platform transcriptomic datasets, and machine learning algorithms. By combining network toxicology, molecular docking, immune infiltration analysis, and regulatory network construction, we systematically identified and validated core genes driving BPA‐induced CD risk.

By intersecting BPA‐associated targets with CD‐related genes, we identified 65 overlapping genes, highlighting key pathways, such as apoptosis, MAPK signaling, and immune–inflammatory regulation. Functional enrichment and network analyses further underscored the centrality of these genes in processes critical to CD pathogenesis, including cytokine‐mediated inflammation, phosphodiesterase activity, and immune cell activation. BPA has been shown to induce inflammatory responses in experimental models; in DSS‐induced murine colitis treated with BPA, levels of IL‐1α, IL‐12p, IL‐13, and IL‐31 were elevated [44]. Similarly, CD‐1 mice exposed to BPA exhibited significantly increased serum levels of TNF‐α, IL‐6, IL‐1β, and IL‐18 [47]. Clinical studies further support a positive correlation between BPA exposure and elevated serum pro‐inflammatory cytokines (e.g., TNF‐α, IL‐6, IL‐23, and IL‐17) in CD patients [24, 48]. Schmitt et al. [49] emphasized the pivotal role of IL‐23 in maintaining and expanding the Th17 lineage through a positive feedback loop involving IL‐17, RORγt, TNF, IL‐1, and IL‐6. This mechanism is implicated in the expansion of pathogenic Th17 cells in CD. GO and network analyses highlighted phosphodiesterase activity as a key pathway. Prior studies show that pro‐inflammatory cytokines, such as TNF‐α and IL‐17, are overexpressed in CD, and PDE4 inhibition elevates cAMP levels, suppressing immune cell activation (e.g., macrophages and T cells) and reducing inflammatory mediator release [50]. Preclinical evidence further supports the therapeutic potential of PDE4 inhibitors, which ameliorate intestinal inflammation in experimental colitis models [51, 52]. In a phase II trial for UC patients, apremilast, an oral PDE4 inhibitor, showed significant clinical improvement. The 30 mg dose of apremilast showed potential for short‐term clinical remission, endoscopic improvement, and a reduction in inflammatory markers in UC treatment, with good long‐term efficacy [53].

Notably, machine learning identified HGF, IL1R1, MMP1, MMP2, and NTRK2 as core mediators of BPA–CD interactions, supported by robust diagnostic performance across multiple datasets. While the high discriminatory performance observed in both the training and validation datasets is encouraging, it should be interpreted cautiously. This performance may reflect the central biological roles of the selected genes in CD pathogenesis, but it may also be influenced by residual batch effects among public datasets, disease subtype homogeneity, or limited sample sizes. Accordingly, the generalizability of the model requires further validation in larger, multicenter clinical cohorts. HGF exhibits dual regulatory roles in IBD pathogenesis. Clinical studies show significantly elevated serum HGF levels in active CD patients, positively correlating with disease activity indices [54]. While HGF exerts protective effects through epithelial proliferation and mucosal regeneration, its overexpression under chronic inflammatory conditions may paradoxically exacerbate inflammation and fibrotic progression. Mechanistically, HGF‐MET signaling drives Th17 cell differentiation via neutrophil‐mediated IL‐1β secretion [55], a critical pathway given the central role of Th17‐mediated inflammation in CD pathogenesis [56]. IL1R1 is multifaceted in its associations with CD. Genetic analyses implicate IL1R1 polymorphisms (rs13015714 and rs2058660) in increased disease susceptibility [57]. Notably, ileal tissues from complex CD cases exhibit upregulated IL1R1 mRNA expression, correlating with apoptosis resistance and senescence‐associated secretory phenotypes, suggesting its involvement in ileal fibrogenesis [58]. In pediatric CD management, exclusive enteral nutrition (EEN) achieves remission in 60%–80% of cases within 6–8 weeks, solidifying its role as a first‐line therapy [59]. Proteomic profiling identifies elevated baseline MMP1 levels in CD patients compared to healthy controls, with marked reduction post‐EEN [60]. This modulation likely occurs through neutrophil and Th17‐related pathways [61, 62]. Emerging evidence highlights MMP2 as a hub gene potentially linked to intestinal fibrotic strictures in CD [63]. In *Gobiocypris rarus*, BPA significantly upregulated MMP1 and MMP2 protein levels in testicular tissue. This upregulation is associated with the activation of cytokines, such as TNF‐α and IL‐1β, along with the activation of the JNK signaling pathway. Activation of this pathway leads to increased levels of MMP1 and MMP2 proteins, which degrade the extracellular matrix and tight junction proteins, disrupting cellular barrier function [64]. While research on NTRK2 remains limited, preliminary data suggest that fecal miR‐16‐5p upregulation in CD patients may target NTRK2 expression [65]. Additionally, BPA directly inhibits BDNF transcription by upregulating miR‐204 expression. BDNF, a ligand for NTRK2, is reduced, leading to decreased activity of the NTRK2 signaling pathway, which inhibits the PI3K/Akt/mTOR pathway, triggering autophagy and inhibiting granulosa cell proliferation [66]. BPA can also significantly reduce NTRK2 phosphorylation levels, leading to decreased expression of synaptic proteins, synaptic dysfunction, and cognitive behavioral deficits. This process may be linked to the inactivation of the NTRK2 signaling pathway [67].

Despite these advances, our study has limitations. First, reliance on publicly available datasets introduces potential batch effects and population biases, though rigorous normalization mitigated these concerns. Second, while molecular docking supports BPA–core gene interactions, experimental validation is necessary to confirm causality. Third, immune infiltration results derived from transcriptomic data require validation through flow cytometry or single‐cell sequencing. Finally, the cross‐sectional nature of the data limits insights into the temporal dynamics of BPA exposure and CD progression.

Our study primarily identifies core genes and pathways associated with the BPA–CD association at the levels of transcriptional regulation and PPI. To further clarify causal relationships and multilevel interaction mechanisms between BPA exposure and CD pathogenesis, future research may proceed in several directions. First, leveraging large‐scale CD GWAS summary statistics, expression quantitative trait locus (eQTL) analyses could be performed for the identified core genes to identify regulatory genetic variants. Mendelian randomization approaches could then be applied to evaluate the causal relationship between genetically predicted gene expression and CD risk, thereby validating the mediating roles of these genes at the genetic level. However, assessing whether BPA exposure modifies these genetic effects is currently not feasible due to the absence of suitable exposure‐related genetic instruments [68, 69]. Second, as a well‐recognized endocrine‐disrupting chemical, BPA has been shown to influence epigenetic modifications, particularly DNA methylation [70–72]. Future studies could integrate publicly available DNA methylation datasets from patients with CD, such as those in GEO or the GWAS Catalog, to examine methylation alterations in BPA‐responsive regions or in promoter regions of the identified core genes, as well as their associations with gene expression and clinical phenotypes. This approach would help elucidate long‐term epigenetic mechanisms linking BPA exposure to CD susceptibility. Finally, multiple studies have demonstrated that BPA can perturb the intestinal microbiota [73–75]. Future research could analyze existing or newly collected metagenomic data from patients with CD to investigate associations between BPA exposure and alterations in microbial composition and functional pathways. Importantly, integrative multiomics analyses could further explore the relationships among microbial metabolite changes, such as short‐chain fatty acids and bile acids, host core gene expression, and immune cell infiltration. This strategy would enable the construction of a comprehensive mechanistic framework linking environmental toxicant exposure, microbiota dysbiosis, host immune and metabolic dysregulation, and disease development

## 5. Conclusion

Our multiomics integration provides robust evidence that BPA exacerbates the risk of CD by dysregulating key genes involved in inflammation, matrix remodeling, and immune homeostasis. These findings establish a critical connection between environmental toxicology and CD pathophysiology, offering valuable mechanistic insights and potential avenues for translational research. Future studies should focus on validating these targets in preclinical models and investigate therapeutic strategies to mitigate the detrimental effects of BPA on gut health.

## Author Contributions

**Liangliang Dai:** conceptualization, data curation, funding acquisition, investigation, project administration, supervision, validation, writing – review and editing. **Chenjie Qiu:** formal analysis, methodology, software, resources, visualization, writing – original draft.

## Funding

No funding was received for this manuscript.

## Ethics Statement

The authors have nothing to report.

## Conflicts of Interest

The authors declare no conflicts of interest.

## Data Availability

Data will be made available upon request.
